# A108 AN EVALUATION OF IMPLEMENTATION OF A CENTRALIZED REFERRAL AND BOOKING PROCESS ON WAIT TIMES FOR COLONOSCOPIES

**DOI:** 10.1093/jcag/gwad061.108

**Published:** 2024-02-14

**Authors:** A Nguyen, G Porwal

**Affiliations:** Cambridge Memorial Hospital, Cambridge, ON, Canada; Cambridge Memorial Hospital, Cambridge, ON, Canada

## Abstract

**Background:**

Colonoscopies in the majority of centres in Ontario are booked by individual endoscopists via their individual offices (practices). As such, wait times for procedures will vary significant from centre to centre, or even from endoscopist to endoscopist at the same centre, depending on individual expertise, experience, procedure types, and local availability of endoscopic resources. This system could lead to significant variability in access to care for patients.

**Aims:**

This study analyzed the effect of implementing a centralized referral and booking process for colonoscopy, for a specific indication, at a single centre in Ontario.

**Methods:**

This was a single centre retrospective study analyzing wait times for patients that underwent a colonoscopy at Cambridge Memorial Hospital (CMH) between April 2017 to July 2022 for either positive fecal occult blood test (FOBT) or fecal immunochemistry test (FIT). The province of Ontario transitioned from FOBT to FIT as the primary tool for colon cancer screening in 2019. Prior to this transition, colonoscopy for positive FOBT performed at CMH were booked by individual endoscopists, with wait times from referral to procedure having great variability. In conjunction with Ontario’s transition to FIT for colon cancer screening, all endoscopists at CMH agreed to participate in a centralized referral and booking process specifically for colonoscopy for positive FIT indication. A chart review of all patients undergoing colonoscopy for either FOBT (non central booking) or FIT (central booking). Patients that self-delayed were excluded. Wait times (WT) was calculated as time between date of referral and date of procedure. The effect of the centralized booking system on wait times was then assessed using statistical analysis.

**Results:**

In total, 344 patients were included for analysis from before March 2019 (non centralized booking process). The average WT in this group was determined to be 99.2 days (range 50-171 days), of which 147 patients had a WT of less than 56 days (42.7%). In contrast, a total 572 patients were included for analysis after March 2019 (centralized booking process), the average WT was determined to be 44.2 days (range 32-59 days), of which, 526 patients had a WT of less than 56 days (92.0%). The centralized booking process reduced average wait times by 55 days (55.4%), and improved procedure WTs meeting mandated time frame of 56 days by 49.3%. This improvement in wait times was most pronounced for endoscopists with the longest wait times in a non centralized system.

**Conclusions:**

The implementation of a centralized referral and booking process (for a single indication for colonoscopy) was associated with significantly reduced WTs. These findings suggest a centralized referral and booking process (for well defined clinical needs) can potentially significantly improve patient wait times.

**Funding Agencies:**

None

